# Parafoveal Word Frequency Does Not Modulate the Effect of Foveal Load on Preview in Chinese Reading: Evidence from Eye Movements

**DOI:** 10.3390/brainsci14040360

**Published:** 2024-04-04

**Authors:** Yue Sun, Sainan Li, Yancui Zhang, Jingxin Wang

**Affiliations:** 1 Faculty of Psychology, Tianjin Normal University, Tianjin 300387, China; sunyuepsy@126.com; 2 Tianjin Academy of Educational Sciences, Tianjin 300191, China; lisainan061@163.com; 3 College of Humanities, Tianjin Agricultural University, Tianjin 300384, China; zhang_yancui@126.com

**Keywords:** foveal load effect, preview, frequency, Chinese reading

## Abstract

The foveal load effect is one of the most fundamental effects in reading psychology, and also one of the most controversial issues in recent years. The foveal load effect refers to the phenomenon that the difficulty of foveal processing affects parafoveal preview. In Chinese reading, whether the foveal load effect exists, as well as whether this effect is modulated by parafoveal word frequency, remains unclear. In this study, the eye-tracking technique was used to track the eye movements of 48 subjects. Utilized the boundary paradigm with single-character words as parafoveal words, the present study manipulated foveal word frequency (high and low), parafoveal word frequency (high and low), and two types of preview (identical preview and pseudocharacter preview) to investigate these questions. The results revealed that the foveal word frequency does not influence preview, suggesting the absence of the foveal load effect when using single-character words as parafoveal words. Furthermore, parafoveal word frequency does not modulate the effect of the foveal load on the preview. This empirical evidence contributes to refining the understanding of the Chinese reading model.

## 1. Introduction

During reading, the reader’s visual field can be categorized into three regions: the foveal region, the parafoveal region, and the peripheral region. The foveal region is the area within 2° of the center of the visual field, which has the highest visual acuity and the greatest ability to discriminate details; the area 2–5° outside the fovea is the parafoveal region, where visual acuity decreases significantly with increasing distance from the fovea, but some information can be obtained; and the peripheral region is the area outside the parafovea [1]. Consequently, readers not only extract information from the foveal region they are currently fixating on but also from the unfixed parafoveal region. This phenomenon of pre-processing information without fixation is called parafoveal preview [2,3]. Numerous studies have demonstrated that preview plays an important role in text processing, leading to a reduction in processing time, and the reduced processing time is called preview benefit [2,3].

**Figure 1:**
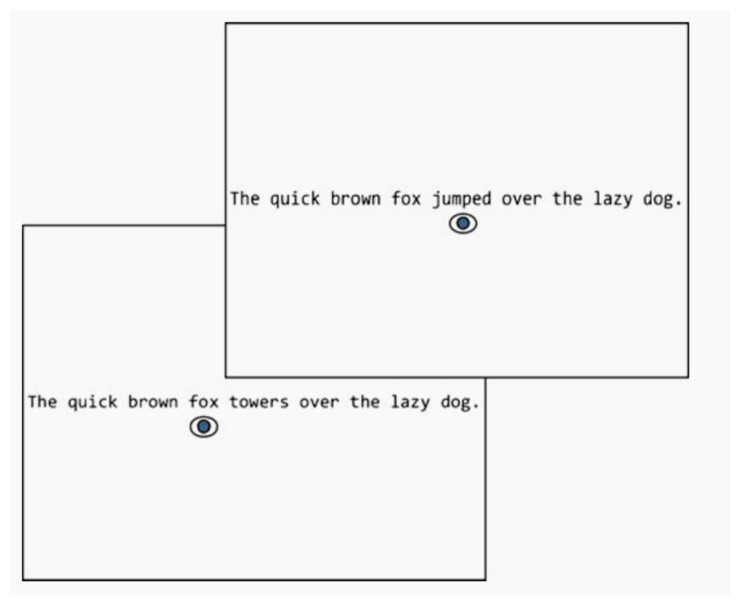
Example of the boundary paradigm [1]. When the subject’s eyes cross an invisible boundary before a critical word in the sentence, it changes from the preview (“towers”) to the target (“jumped”).

Eye-tracking technology can track the reader’s eye movements, making it possible to analyze the reader’s cognitive activity during the reading process. We often use the boundary paradigm to study the preview effect, examining parafoveal processing by manipulating whether or not effective information can be obtained in the preview [1,2]. In the boundary paradigm, there is an invisible boundary between foveal words and parafoveal words, and once the readers’ eyes cross the boundary, the preview word becomes the target word (Figure 1). Before then, the preview word was replaced by some form of preview, so that the relationship between the preview word and the target word (orthographical, phonological, semantic relevant, or irrelevant) could be controlled to explore what kind of processing, and to what extent, the parafoveal word was processed before fixation [1,2].

The foveal load effect, a phenomenon in which the difficulty of foveal processing affects the parafoveal preview, was initially proposed by Henderson and Ferreira (1990) [4]. In their experiments, they found that increasing the difficulty of the foveal word may reduce the preview benefit, giving rise to the “foveal load hypothesis”. Once proposed, the foveal load hypothesis was incorporated into the theoretical framework of the two most influential models of eye movement control, the E-Z reader model and the SWIFT model. Although the two models’ predictions of the foveal load effects go in opposite directions, a foveal load mechanism is constructed in their architectures, modeling the modulation of parafoveal preview by the foveal load [5,6,7,8,9]. The Chinese reading model (CRM) proposed by Li et al. (2020) [10], based on the interactive activation model, also simulated the foveal load effect, and the direction is consistent with the E-Z reader model. This evidence showed that the foveal load effect is one of the most basic effects in reading.

The foveal load effect has also been one of the most controversial issues in recent years. Among the related studies on alphabetic language, only some of them replicated the experimental results of Henderson and Ferreira (1990) [11,12,13,14], while the others did not obtain the foveal load effect or only partially obtained the effect [15,16,17,18,19,20,21,22]. One possible reason is that the high variability of word length in alphabetic language, especially the different word lengths of the parafoveal word may be an important reason for the inconsistent results [23,24,25,26]. Compared to short words, long words extend to a larger range of the parafoveal region, and thus, preview inevitably varies across experimental materials and experiments [26]. In contrast, using Chinese to study the foveal load effect has the advantage, that Chinese is character-based with each character occupying the same space, and with a relatively concentrated distribution of word lengths (72% for two-character words and 6% for single-character words), and thus, the factor of word length can be well controlled and manipulated during Chinese reading, enabling the exploration of preview benefits within a fixed processing range.

In Chinese reading, related studies are scarce, and studies using single-character words as parafoveal words have failed to observe the foveal load effect. Zhang et al. (2019) [23] manipulated foveal word frequency (high and low) and two types of preview (identical preview, and pseudocharacter preview) to investigate the foveal load effect in Chinese reading with the boundary paradigm. Their study employed single-character words as parafoveal target words, and restricted preview manipulations to single-character words. However, they did not obtain the interaction between foveal load and parafoveal preview, indicating that the preview benefit remained unaffected regardless of the foveal word load. Zhang et al. (2020a) [24] used the same experimental materials as Zhang et al. (2019) [23] (the parafoveal was also single-character words) and added the variable of fast readers and slow readers to the existing studies, and the results also did not obtain the modulated effect of foveal load on preview, which is in line with the results of the Zhang et al. (2019) [23].

Does the foveal load effect not exist when the parafoveal word is a single-character word? Additionally, is there any factor that may modulate this effect? Previous studies have shown that parafoveal preview is influenced by the properties of the parafoveal word, especially the frequency of the parafoveal word [1,27]. Consequently, do the properties of the parafoveal word influence the effect of foveal load on preview? In other words, do the properties of the parafoveal word play an important role in modulating the foveal load effect? The modulating role of parafoveal processing load (word frequency) on the foveal load effect has been investigated in alphabetic language. Kennsion et al. (1995) [17] examined whether working memory affected the preview effect, and whether preview was modulated by the difficulty of the foveal and the parafoveal word. In addition to categorizing subjects into high and low working memory groups, the study also manipulated foveal word frequency (high and low), parafoveal word frequency (high and low), and two types of preview (identical preview and pseudoword preview). The result showed that neither the effect of working memory on the preview effect nor the interaction between foveal word frequency and preview type was observed. However, a three-way interaction was found on parafoveal word frequency, foveal word frequency, and preview type, which indicated that the foveal load effect exists only when the parafoveal was difficult to process (for low-frequency words), that is the difficulty of parafoveal processing modulated the foveal load effect. Using a boundary paradigm, Schroyens et al. (1999) [14] similarly investigated the factors that influence the foveal load effect. Schroyens et al. (1999) manipulated the word frequency of the foveal (high, low, and z-letter strings), the length of the foveal words (three letters, five letters), the frequency of the parafoveal word (high and low), and two types of preview (identical preview, masked). They found the interaction between the foveal word frequency and preview on gaze duration, indicating that the preview benefit was smaller when the foveal was a low-frequency word compared to high-frequency words and z-letter strings. The experiment did not find a three-way interaction between parafoveal word frequency, foveal word frequency, and preview type. However, specific comparisons revealed that the foveal load effect is present only for low-frequency parafoveal words, aligning with the findings of Kennison et al. (1995) [17]. The findings showed that parafoveal word frequency plays an important role in modulating the foveal load effect in alphabetic language. 

Due to the special characteristics of Chinese, it is unknown whether the parafoveal word frequency affects the foveal load effect in Chinese reading. Moreover, there has been no research to explore the factors that influence the foveal load effect in Chinese reading so far. Thus, the current study aimed to investigate the existence of the foveal load effect in Chinese reading when using single-character words as parafoveal words, and whether it is modulated by the difficulty of the parafoveal processing. Referring to Kennsion’s (1995) study, word frequency was chosen as the processing difficulty of the parafoveal (high-frequency word: easy to process; low-frequency word: difficulty to process), and manipulated three factors, foveal word frequency (high and low), parafoveal word frequency (high and low), and two types of previews (identical preview and pseudocharacters). Previous studies have shown that potential disruptive effects of “abnormal” baselines, leading to processing costs that may influence the foveal load effect [16,21,22]. Therefore, the present study used orthographically legal pseudocharacters (in which the correct radicals are placed in the correct position but the combination is not a true character) as the baseline to minimize the interference caused by parafoveal masking. To be clear, the pseudocharacter has no orthographical, phonological, or semantic connection to the target word. Based on the results from previous studies [23,24], we predicted that there is no interaction between foveal word frequency and preview type when using single-character words as the parafoveal words, but there may be a three-way interaction between parafoveal word frequency and foveal word frequency and preview type.

## 2. Materials and Methods

### 2.1. Ethical Approval

The present study was approved by the Research Ethics Committee of the Faculty of Psychology, Tianjin Normal University (Approval Number, 2024011202), and conducted by the principles of the Declaration of Helsinki. All the participants signed informed consent and volunteered to participate in the experiment.

### 2.2. Participants

We used MorePower 6.0.4 software [28] to calculate the subject size of this experiment. When the effect size reached *f* = 0.42 (close to prior research of Zhang et al., 2019) and the significance level reached a = 0.05, 48 subjects were needed to achieve the power of 0.8. Thus, 48 college students were selected with a mean age of 19.9 years (*SD* = 2.07), who had normal naked eye vision or corrected vision. All of them were native speakers of Chinese, and none of them were aware of the purpose of the experiment. They were paid 30 RMB at the end of the experiment. 

### 2.3. Experimental Design

A three-factor within-subjects design of 2 (foveal word frequency: high, low) × 2 (parafoveal word frequency: high, low) × 2 (type of preview: identical preview, pseudocharacter preview) was used.

### 2.4. Experimental Materials

**Figure 2:**
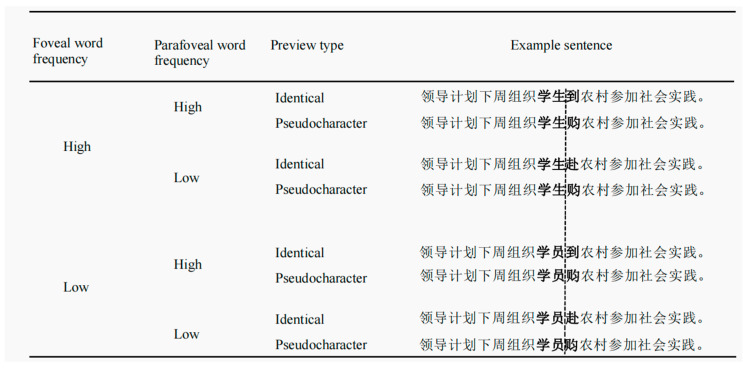
An example of experimental material (This is the translation of “领导计划下周组织学生/学员到/赴农村参加社会实践”: Leaders are planning to organize a social trip to the countryside for students/trainees next week). The high- and low-frequency word pairs in the fovea are “students” (学生) and “trainees” (学员). The high- and low-frequency word pairs in the parafovea are “到” and “赴”, which both mean “go to”). The dotted line represents the invisible boundary. The two-character word bolded before the boundary is the foveal word, and the single-character word bolded after the boundary is the parafoveal word. As readers’ eyes cross the invisible boundary, the preview word is replaced by the target word (“到”/“赴”).

To minimize skipping and ensure parafoveal word preview occurs on foveal words, two-character words were utilized as foveal words, paired with single-character words as parafoveal words. A total of 80 pairs of high- and low-frequency word pairs for both foveal and parafoveal words were selected from the word frequency corpus of Cai et al. (2010) [29] and were coded in the same sentence frame. Figure 2 shows an example of experimental material. Foveal high-frequency words had significantly higher word frequency than low-frequency words (*F* (1, 158) = 9.44, *p* = 0.002, high-frequency words ranged from 45.28 to 11,080.02 per million, low-frequency words ranged from 0.09 to 10.64 per million), and there was no significant difference in the number of strokes between high- and low-frequency foveal words pairs (*F* (1, 158) = 0.46, *p* = 0.50). The parafoveal high-frequency words had significantly higher word frequency than low-frequency words (*F* (1, 158) = 24.97, *p* < 0.001, high-frequency words ranged from 43.82 to 12811.05 per million and low-frequency words ranged from 0.42 to 20.12 per million), and there was no significant difference in the number of strokes for high- and low-frequency parafoveal word pairs (*F* (1, 158) = 3.01, *p* = 0.09). Both foveal high- and low-frequency words were low-predictability words with no significant predictability difference (*F* (1, 158) = 1.79, *p* = 0.18). Similarly, the parafoveal word was a low-predictability word in four conditions, and there was no predictability difference across the four conditions (*F* (3, 316) = 1.05, *p* = 0.37). All sentences were highly fluent (1 for very unnatural, 7 for very natural), and there was no significant fluency difference between the four conditions (*F* (3, 316) = 0.45, *p* = 0.72), with means and standard deviations reported in Table 1.

The type of preview comprised two levels, identical preview and pseudocharacter preview, resulting in 80 groups of sentences with eight experimental conditions each. Sentences with different conditions in each group were distributed across 8 blocks using a Latin square design. Each block included 6 practice sentences and 40 filler sentences, totaling 126 sentences per block. Additionally, 30% of the sentences were followed by reading comprehension judgment questions. 

### 2.5. Experimental Apparatus

The Eyelink 1000 plus eye-tracking device, manufactured by SR Research, served as the experimental apparatus, featuring a sampling rate of 1000 Hz. The subject’s screen had a refresh rate of 144 Hz and a resolution of 1920 × 1080 pixels. The subject’s eyes were positioned 60 cm from the screen. The font used for the material was No. 32 Song, with each word on the screen approximately 42.5 × 42.5 pixels in size, providing each word with a viewing angle of approximately 1.12°.

### 2.6. Experimental Procedure

In the eye movement experiment, each subject was administered individually. Before the experiment, the subjects were first asked to read and sign the informed consent form, and then were guided to sit in front of the subject machine and told to place their chin on the chin rest, as much as possible to avoid head movements during the experiment. Afterwards, the instructions were presented on the screen of the subject machine, and the subjects were told to read the sentences appearing on the screen carefully from left to right, then to press the space bar to read the next sentence after finishing reading. Occasionally, questions assessing semantic understanding followed certain sentences, prompting subjects to respond. After ensuring that the subjects understood the experimental task, the subjects’ eyes were calibrated at three points, and then the practice session began. Once familiar with the process, the formal experiment commenced. The experiment was calibrated at three-point intervals of four sentences, and a “+” was placed at the beginning of each sentence to ensure that subjects started reading at the beginning of the sentence. The entire experiment lasted approximately 30 min. After it was over, subjects were asked if they had perceived a change and assessed the number of sentences that had changed.

## 3. Results

Four subjects who were aware of most changes were excluded, resulting in 48 valid subjects. Subjects exhibited a reading judgment correct rate of 92.7%, indicating careful comprehension of the experiment’s sentences. Before analyzing the eye movement data, we removed any fixations longer than 1200 ms or shorter than 80 ms. Trials were then excluded in which: (1) trials with lost tracking due to subjects’ head movements (approximately 0.91% of total trials); (2) trials with sentence fixation points less than or equal to 5 (about 1.54% of total trials); (3) trials with (a) screen changes too late (delayed boundary changes) (about 12.03% of total trials); (b) trials in which the boundary changed too early (about 0.91% of total trials); (c) trials in which the boundary was mistakenly triggered by eye “Hooks” (about 4.64% of total trials); (d) trials in which the boundary was incorrectly triggered due to saccade (about 0.13% of total trials); (e) trials in which the boundary was incorrectly triggered due to blinking (about 0.83% of total trials). In total, 20.99% of trials were deleted, leaving 79.01%.

The main measures of eye movements included the following: (1) First fixation duration (FFD) is the duration of the first fixation point that enters an interest area for the first time in the first-pass reading, reflecting the characteristics of the earliest lexical processing. (2) Single fixation duration (SFD) is the duration of gaze when there is one and only one fixation within an interest area in the first-pass reading, which reflects the process of early lexical recognition. (3) Gaze duration (GD) is the duration from the beginning of the first fixation to the first time the fixation point leaves the current interest area in the first-pass reading, reflecting the earlier lexical processing. (4) Skipping probability (SP) refers to the rate of skipping an interest area in the first-pass reading, reflecting the situation that the target word is processed in advance, and words that are easily processed are likely to be skipped. (5) Forward saccade length (FSL) refers to the distance between the position of the fixations launched from the pre-target words to the position of the later words, reflecting the difficulty of the current processing, with longer forward saccade lengths indicating easier processing [30].

To analyze the data, we constructed linear mixed models (LMMs) by using the lem4 package in R 4.1.2 (R Core Team, 2021) and RStudio (version number: 2021.09.01, 2021). Fixation durations and forward saccade length were log-transformed before statistical analyses and data beyond three standard deviations were removed. Skipping probability rates were analyzed using logistic LMMs. The model incorporated each variable and their interactions as fixed factors while encompassing cross-random effects of both participants and items. In instances where the most comprehensive random effects model failed to converge, a stepwise simplification approach was adopted. The log-transformed analysis and untransformed analysis of fixation duration measures obtained the same significance pattern. Across all analysis results, a *t*/*z* value greater than 1.96 indicated a significant difference (*p* < 0.05).

### 3.1. Analyses of the Foveal Word Region

The means and standard deviations for each eye movement measure of the foveal region are reported in Table 2, and the fixed-effects estimates are reported in Table 3. The fixation rate for foveal words was 79%, indicating that the use of two-character words for foveal was less likely to be skipped and that the preview data obtained were valid. 

The main effect of foveal word frequency was significant on all fixation times and skipping probability (fixation times: *t*s > 6.56, *p*s < 0.001; skipping probability: *z* = −3.22, *p* = 0.001). High-frequency words cause shorter fixations than low-frequency words and are skipped more often than low-frequency words, suggesting a stable word frequency effect, and the manipulation of foveal word frequency was valid. The main effect of the preview type was not significant on all fixation times and skipping probability (fixation times: |*t*|s < 1.25, *p*s > 0.05; skipping probability: *z* = −0.76, *p* = 0.445). There was a main effect of parafoveal word frequency on gaze duration (*t* = 2.17, *p* = 0.03), and the three-way interaction on FFD and GD was also significant (*t*s > 2.29, *p*s < 0.05), indicating the presence of a parafoveal-on-foveal effect of word frequency, which represents the influence of parafoveal processing on foveal. Additionally, the main effects of foveal word frequency, parafoveal word frequency, and preview type were all significant on forward saccade length (|*t*|s >3.07, *p*s < 0.01), indicating longer forward saccade lengths for foveal high-frequency words than for low-frequency words, longer for parafoveal high-frequency words than for low-frequency words, longer for parafoveal identical preview than for pseudocharacter preview. Two-way interaction between parafoveal word frequency and preview type was significant (*t* = 3.27, *p* = 0.001), indicating that when under identical preview, the forward saccade length was longer for high-frequency parafoveal words compared to low-frequency words, but when under pseudocharacter preview, there was no significant difference in forward saccade length between high- and low-frequency parafoveal words. However, the three-way interaction was not significant. The results in forward saccade length indicated that both foveal and parafoveal processing independently influenced forward saccade length, whereas parafoveal word frequency and preview type together influenced the forward saccade length.

### 3.2. Analysis of the Parafoveal Word Region

**Figure 3:**
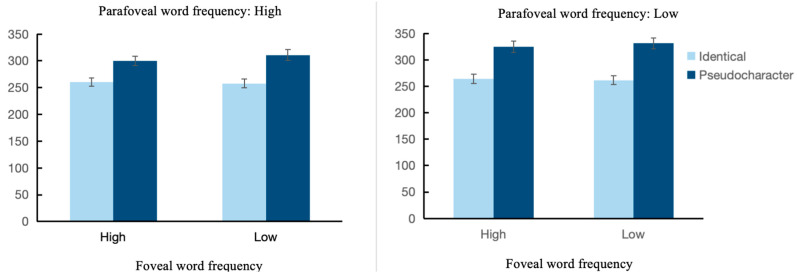
No interaction between foveal word frequency and preview type (using GD as an example), indicating the absence of the foveal load effect. Neither was the three-way interaction.

The means and standard deviations for each eye movement measure of the parafoveal region are reported in Table 4, and the fixed-effects estimates are reported in Table 5. As shown in the table, the main effect of foveal word frequency was not significant on all fixation times (|*t*|s < 0.51, *p*s > 0.05), indicating that there was no spillover effect, that foveal processing load did not spillover from the foveal word to the upcoming parafoveal word. However, the main effect of foveal word frequency was significant on skipping probability, indicating that high-frequency foveal words cause more skipping of parafoveal words than low-frequency foveal words. The main effect of parafoveal word frequency was not significant on all fixation times and skipping probability (fixation times:|*t*|s < 1.84, *p*s > 0.05; skipping probability: *z* = −1.38, *p* = 0.167), but there was a marginal significance on gaze time (*t* = 1.83, *p* = 0.067), indicating shorter processing time when the parafoveal word was of high frequency compared to low frequency. This, combined with the parafoveal-on-foveal effect in previous analyses of the foveal word region, suggests that the manipulation of parafoveal word frequency was equally effective. Preview type was significant on all fixation times and skipping probability (fixation times: *t*s > 6.67, *p*s < 0.001; skipping probability: *z* = −6.38, *p* < 0.001), that shorter processing times for identical preview than for pseudocharacter preview, and a higher skipping probability for identical preview than for pseudocharacter preview, indicating that the control for preview type was effective. There was no interaction between foveal word frequency and preview type across all fixation times and skipping probability (fixation times: *t*s < 0.88, *p*s > 0.05; skipping probability: *z* = −0.90, *p* = 0.37), indicating that foveal load did not modulate preview benefits when the parafoveal was a single-character word, which is consistent with previous findings [23,24]. In addition, the three-way interaction between foveal word frequency, parafoveal word frequency, and preview type was similarly nonsignificant on all fixation times and skipping probability (|*t*|s < 0.43, *p*s > 0.05; skipping probability: *z* = 1.07, *p* = 0.28), indicating that when the parafoveal is a single-character word, the word frequency of the parafoveal does not modulate the effect of foveal load on the preview (refer to Figure 3). No other two-way interaction was observed.

## 4. Discussion

The present study investigated the existence of the foveal load effect when using single-character words as parafoveal words, and whether it is modulated by the frequency of parafoveal words in Chinese reading. In the experiment, the fixation rate to the foveal words was 79%, indicating that using two-character words as the foveal word was less likely to be skipped and the preview data obtained were valid. The main effects of foveal word frequency and parafoveal preview type were significant in the foveal and parafoveal region analyses, respectively, indicating that the manipulation of foveal word frequency and parafoveal preview type was valid. Although the main effect on parafoveal word frequency was not obtained for parafoveal region analyses, there was a parafoveal-on-foveal effect in the foveal region analyses, which similarly suggests that the manipulation of parafoveal word frequency was equally valid. More importantly, the results showed there was no modulating effect of foveal load on preview when the parafoveal was a single-character word, which is in line with the results of previous studies [23,24]. 

Various eye movement control models have incorporated the role of foveal load on preview. The E-Z reader model, based on sequential processing, assumes that attention is shifted sequentially between words, and the longer the foveal word is processed, the later the shift in attention to the parafoveal, whereas the time of saccade-planning is fixed, so that less time is spent processing the parafoveal word, and the preview benefit is reduced [5,6]; whereas the SWIFT model argues that attention is parallel distributed within perceptual span, so as the foveal processing time increases, adjacent parafoveal words will also be processed more, and the preview benefit will be greater [7,8,9]. In Chinese reading, the Chinese reading model (CRM) [10] based on the interactive activation model, also simulates the foveal load effect with the word frequency as the foveal load, arguing that words that can be formed from Chinese characters within the perceptual span are activated, competing with each other for the only winner. Moreover, when the activation of words within a slot is greater than a certain level (0.3), the word frequency of the foveal word affects the processing of the parafoveal words (more processing of parafoveal words when the foveal word is a high-frequency word), which is in the same direction as that predicted by the E-Z reader model. Thus, all three models assume that foveal load influences preview. However, the present study failed to obtain a modulating effect of foveal load on preview when the parafoveal preview was confined to the single-character spatial range, potentially due to Chinese language characteristics. 

Since Chinese is closely aligned, more information can be obtained in a limited space, and more preview can be obtained on parafoveal words, with a greater preview benefit [31,32]. Therefore, Zhang et al. (2020a) argued that the absence of the foveal load effect when using single-character words as the parafoveal words can be explained by the fact that when the preview manipulation is restricted to single-character words closest to the foveal, the preview words are adjacent to the foveal words, and thus, considerable preview benefits can be obtained on the parafoveal words regardless of the foveal load, and thus, no difference in preview benefits can be observed. Moreover, it has been shown that the foveal load effect exists when using two-character words as parafoveal words [33,34]. Therefore, the foveal load effect may reflect the spatial “accumulation” of difference in preview, that the degree of reduction in processing efficiency of the parafoveal word varies with the load of the foveal word, and thus, it is possible to observe differences in the amount of preview by expanding the extent of the preview manipulation. 

Furthermore, the three-way interaction between foveal word frequency, parafoveal word frequency, and preview type was not significant, which indicated that when the parafoveal was a single-character word, the word frequency of the parafoveal did not modulate the effect of foveal load on preview, which is diverge from prior research on alphabetic languages [14,17]. Moreover, we did not observe the interaction between parafoveal word frequency and preview type, which is similarly with previous study [35]. This absence of the interaction between parafoveal word frequency and preview type implies that parafoveal word frequency does not influence preview benefit, and might explain why parafoveal word frequency fails to modulate the effect of foveal load on preview. 

Additionally, a parafoveal-on-foveal effect of word frequency on gaze time was observed, indicating that the word frequency of the parafoveal influences the processing of foveal words. This phenomenon might be attributed to the compact nature of Chinese compared to alphabet languages. Given that the parafoveal is a single-character word, closest to the foveal, when the fixation on the foveal word is near the boundary, it is easy to bring the parafoveal word into the foveal field, affecting the foveal processing. Moreover, the Chinese reading model assumes that words that can be formed from Chinese characters within the perceptual span are activated and compete with each other for the only winner, suggesting the possibility of the existence of the parafoveal-on-foveal effect. Moreover, regarding forward saccade length, both foveal and parafoveal affected forward saccade length, but there was no interaction. It was shown that the forward saccade length was longer when the foveal was easy to process (for low frequency), and the more preview information the parafoveal word received (identical preview), the longer the forward saccade length was, which is consistent with the previous studies [23,24,36]. Additionally, the joint influence of parafoveal word frequency and preview type on forward saccade length supported the Chinese reading model’s prediction that more information about a Chinese character in the parafoveal leads to longer forward saccade lengths [10]. Therefore, although the foveal load effect is not obtained, most of the results of the experiment can be explained by the Chinese reading model.

However, there are limitations to the current study. Firstly, the study is using college students as a proxy for normal adults. The college student group has more time for reading during the study compared with other adults. Therefore, there are limitations in the generalization of the findings. Secondly, the findings based on normal adults may not necessarily be generalized to children and older people. This issue could be explored in subsequent studies with children and older adults. Thirdly, the study used word frequency as the foveal load. It has been shown that the foveal load at different processing levels has different effects on preview [4,12,22]. Consideration could be given to using other foveal loads to investigate this issue.

In summary, no foveal load effect on preview was observed when constraining the parafoveal word to a single-character space, and the parafoveal word frequency did not modulate this effect. These findings, not currently explained by existing reading models, may be linked to the characteristics of Chinese. Consequently, the results of the experiment provide empirical evidence for the Chinese reading model.

## 5. Conclusions

In Chinese reading, when using single-character words as parafoveal words, the foveal word frequency does not affect the preview benefit, suggesting the absence of the foveal load effect, and the frequency of the parafoveal word does not modulate the effect of foveal load on preview. The results of this study provide empirical evidence for the Chinese reading model.

## Figures and Tables

**Table 1:** Basic information of sentences with different foveal load and parafoveal load (*SD*s in parentheses).

	Foveal Word Frequency (Per Million)	Foveal Word Stroke	Foveal Word Predictability (%)	Parafoveal Word Frequency (Per Million)	Parafoveal Word Stroke	Parafoveal Word Predictability (%)	Sentence Naturalness
High- frequency	475.72 (1337.74)	16.11 (4.66)	0.03 (0.06)	1641.14 (2922.18)	8.61 (2.63)	HH: 0.02 (0.06)	HH: 5.71 (0.53)
						HL: 0.01 (0.04)	HL: 5.71 (0.65)
Low- frequency	3.80 (2.78)	16.60 (4.43)	0.02 (0.05)	8.50 (5.80)	9.41 (3.13)	LH: 0.02 (0.06)	LH: 5.81 (0.61)
						LL: 0.01 (0.04)	LL: 5.74 (0.60)

Note. HH: high-frequency of both foveal word and parafoveal word; HL: high-frequency foveal word and low-frequency parafoveal word; LH: low-frequency foveal word and high-frequency parafoveal word; LL: low-frequency foveal word and low-frequency parafoveal word.

**Table 2:** Eye movement measures for the foveal word region across conditions (*SD*s in parentheses).

Foveal Word	High Frequency				Low Frequency			
Parafoveal Word	High Frequency		Low Frequency		High Frequency		Low Frequency	
Preview Type	Identical	Pseudocha.	Identical	Pseudocha.	Identical	Pseudocha.	Identical	Pseudocha.
Skipping probability	0.25 (0.43)	0.24 (0.43)	0.22 (0.41)	0.24 (0.43)	0.19 (0.39)	0.17 (0.38)	0.21 (0.41)	0.20 (0.40)
First fixation duration (ms)	222.93 (77.31)	225.19 (67.67)	232.73 (83.22)	223.49 (80.22)	250.05 (90.85)	236.56 (81.13)	247.40 (83.86)	255.81 (98.74)
Single fixation duration (ms)	221.78 (75.67)	223.73 (65.63)	229.06 (75.26)	220.48 (73.44)	247.61 (89.86)	234.82 (81.64)	248.75 (84.59)	250.38 (94.90)
Gaze duration (ms)	234.85 (98.41)	243.30 (100.29)	254.44 (116.98)	245.67 (128.47)	294.89 (150.00)	279.00 (155.04)	288.71 (151.57)	310.89 (174.68)
Forward saccade length (character)	2.09 (0.96)	1.77 (0.81)	1.99 (0.96)	1.78 (0.75)	2.00 (0.95)	1.64 (0.72)	1.78 (0.86)	1.65 (0.83)

Note. Pseudocha. = pseudocharacter.

**Table 3:** Fixed-effects estimates from the linear mixed-effects models and 95% confidence intervals for the eye movement measures at the foveal word.

Fixed Effect	*b*	CI	*SE*	*t/z*	*p*
First fixation duration					
Foveal word frequency	**0.08**	**[0.06, 0.10]**	**0.01**	**6.57**	**<0.001**
Parafoveal word frequency	0.02	[−0.00, 0.04]	0.01	1.61	0.107
Preview type	−0.01	[−0.04, 0.02]	0.01	−0.83	0.411
Foveal word frequency × Parafoveal word frequency	0.02	[−0.02, 0.07]	0.02	0.98	0.329
Foveal word frequency × Preview type	−0.01	[−0.06, 0.04]	0.02	−0.38	0.702
Parafoveal word frequency × Preview type	0.02	[−0.03, 0.06]	0.02	0.64	0.521
Foveal word frequency × Parafoveal word frequency × Preview type	**0.11**	**[0.02, 0.21]**	**0.05**	**2.30**	**0.021**
Single fixation duration					
Foveal word frequency	**0.09**	**[0.06, 0.11]**	**0.01**	**6.67**	**<0.001**
Parafoveal word frequency	0.02	[−0.01, 0.04]	0.01	1.55	0.122
Preview type	−0.02	[−0.05, 0.01]	0.01	−1.24	0.224
Foveal word frequency × Parafoveal word frequency	0.04	[−0.01, 0.09]	0.03	1.46	0.145
Foveal word frequency × Preview type	−0.02	[−0.07, 0.03]	0.03	−0.79	0.433
Parafoveal word frequency × Preview type	0.00	[−0.05, 0.05]	0.03	0.03	0.976
Foveal word frequency × Parafoveal word frequency × Preview type	0.09	[−0.01, 0.19]	0.09	1.76	0.079
Gaze duration					
Foveal word frequency	**0.15**	**[0.12, 0.18]**	**0.02**	**9.85**	**<0.001**
Parafoveal word frequency	**0.03**	**[0.00, 0.06]**	**0.02**	**2.17**	**0.030**
Preview type	−0.00	[−0.03, 0.03]	0.02	−0.21	0.832
Foveal word frequency × Parafoveal word frequency	0.02	[−0.04, 0.08]	0.03	0.61	0.541
Foveal word frequency × Preview type	−0.01	[−0.07, 0.05]	0.03	−0.36	0.717
Parafoveal word frequency × Preview type	0.02	[−0.04, 0.08]	0.03	0.74	0.459
Foveal word frequency × Parafoveal word frequency × Preview type	**0.17**	**[0.05, 0.29]**	**0.06**	**2.71**	**0.007**
Forward saccade length					
Foveal word frequency	**−0.09**	**[** **−0.** **12, −0.06]**	**0.02**	**−5.26**	**<0.001**
Parafoveal word frequency	**−0.04**	**[** **−0.07, −0.02]**	**0.01**	**−3.08**	**0.002**
Preview type	**−0.14**	**[** **−0.** **17, −0.11]**	**0.01**	**−9.87**	**<0.001**
Foveal word frequency × Parafoveal word frequency	−0.03	[−0.09, 0.02]	0.03	−1.19	0.23
Foveal word frequency × Preview type	−0.01	[−0.06, 0.05]	0.03	−0.30	0.76
Parafoveal word frequency × Preview type	**0.09**	**[0.04, 0.15]**	**0.03**	**3.27**	**0.001**
Foveal word frequency × Parafoveal word frequency × Preview type	0.04	[−0.07, 0.15]	0.06	0.72	0.47

Note. Significant effects are indicated in bold. CI = confidence intervals.

**Table 4:** Eye movement measures for the parafoveal word region across conditions (*SD*s in parentheses).

Foveal Word	High Frequency				Low Frequency			
Parafoveal Word	High Frequency		Low Frequency		High Frequency		Low Frequency	
Preview Type	Identical	Pseudocha.	Identical	Pseudocha.	Identical	Pseudocha.	Identical	Pseudocha.
Skipping probability	0.63 (0.48)	0.49 (0.50)	0.58 (0.49)	0.47 (0.50)	0.57 (0.50)	0.41 (0.49)	0.49 (0.50)	0.45 (0.50)
First fixation duration (ms)	251.60 (82.61)	284.16 (106.66)	255.84 (89.31)	298.24 (117.12)	249.92 (99.17)	291.31 (122.06)	247.90 (96.87)	302.99 (134.11)
Single fixation duration (ms)	251.52 (81.69)	286.42 (108.45)	253.90 (87.11)	300.23 (120.67)	251.09 (100.14)	287.59 (118.83)	246.12 (96.40)	309.69 (136.27)
Gaze duration (ms)	260.08 (91.69)	300.21 (119.00)	263.93 (114.95)	324.81 (149.14)	257.58 (105.50)	310.96 (152.14)	261.76 (114.99)	331.28 (153.38)

Note. Pseudocha. = pseudocharacter.

**Table 5:** Fixed-effects estimates from the linear mixed-effects models and 95% confidence intervals for the eye movement measures at the parafoveal word.

Fixed Effect	*b*	CI	*SE*	*t/z*	*p*
First fixation duration					
Foveal word frequency	−0.01	[−0.05, 0.03]	0.02	−0.50	0.621
Parafoveal word frequency	0.02	[−0.01, 0.06]	0.02	1.20	0.230
Preview type	**0.13**	**[0.09, 0.17]**	**0.02**	**6.68**	**<0.001**
Foveal word frequency × Parafoveal word frequency	−0.00	[−0.08, 0.07]	0.04	−0.05	0.964
Foveal word frequency × Preview type	0.03	[−0.04, 0.11]	0.04	0.87	0.385
Parafoveal word frequency × Preview type	0.03	[−0.05, 0.10]	0.04	0.73	0.463
Foveal word frequency × Parafoveal word frequency × Preview type	−0.02	[−0.17, 0.13]	0.08	−0.31	0.761
Single fixation duration					
Foveal word frequency	−0.01	[−0.05, 0.03]	0.02	−0.41	0.685
Parafoveal word frequency	0.03	[−0.01, 0.07]	0.02	1.35	0.176
Preview type	**0.14**	**[0.10, 0.18]**	**0.02**	**6.89**	**<0.001**
Foveal word frequency × Parafoveal word frequency	0.01	[−0.06, 0.09]	0.04	0.34	0.732
Foveal word frequency × Preview type	0.03	[−0.05, 0.11]	0.04	0.82	0.413
Parafoveal word frequency × Preview type	0.05	[−0.02, 0.13]	0.04	1.35	0.178
Foveal word frequency × Parafoveal word frequency × Preview type	0.02	[−0.14, 0.17]	0.08	0.25	0.806
Gaze duration					
Foveal word frequency	−0.00	[−0.05, 0.04]	0.02	−0.12	0.902
Parafoveal word frequency	0.04	[−0.00, 0.08]	0.02	1.83	0.067
Preview type	**0.16**	**[0.12, 0.20]**	**0.02**	**7.85**	**<0.001**
Foveal word frequency × Parafoveal word frequency	0.02	[−0.06, 0.10]	0.04	0.54	0.592
Foveal word frequency × Preview type	0.03	[−0.05, 0.11]	0.04	0.64	0.521
Parafoveal word frequency × Preview type	0.05	[−0.03, 0.13]	0.04	1.27	0.206
Foveal word frequency × Parafoveal word frequency × Preview type	−0.03	[−0.19, 0.13]	0.08	−0.42	0.677

Note. Significant effects are indicated in bold. CI = confidence intervals.

## Data Availability

The data presented in this study are available upon 10.6084/m9.figshare.25264111.
